# Comparative Structure–Property Relationship between Nanoclay and Cellulose Nanofiber Reinforced Natural Rubber Nanocomposites

**DOI:** 10.3390/polym14183747

**Published:** 2022-09-07

**Authors:** Bunsita Wongvasana, Bencha Thongnuanchan, Abdulhakim Masa, Hiromu Saito, Tadamoto Sakai, Natinee Lopattananon

**Affiliations:** 1 Department of Rubber Technology and Polymer Science, Faculty of Science and Technology, Prince of Songkla University, Pattani 94000, Thailand; 2 Rubber Engineering & Technology Program, International College, Prince of Songkla University, Songkhla 90110, Thailand; 3 Department of Organic and Polymer Materials Chemistry, Tokyo University of Agriculture and Technology, Koganei-shi 184-8588, Tokyo, Japan; 4 Organization for Innovation and Social Collaboration, Shizuoka University, 3-5-1 Johoku, Naka-ku, Hamamatsu City 432-8011, Shizuoka, Japan

**Keywords:** natural rubber, nanocomposites, nanoclay, cellulose nanofibers, mechanical property

## Abstract

Natural rubber (NR) nanocomposites reinforced with five parts per hundred rubber (phr) of two different nano-fillers, i.e., nanoclay (abbrev. NC) and cellulose nanofiber (abbrev. CNF), were prepared by using latex mixing approach, followed by mill-compounding and molding. The morphology, stress–strain behavior, strain-induced crystallization, and bound rubber of the NR nanocomposites were systematically compared through TEM, tensile test, WAXS, DMA, and bound rubber measurement. The aggregated CNFs were observed in the NR matrix, while the dispersed nanosized clay tactoids were detected across the NR phase. The reinforcement effects of NC and CNF were clearly distinct in the NR nanocomposites. At the same nano-filler content, the addition of NC and CNF effectively accelerated strain-induced crystallization of NR. The high tensile strength obtained in the NC-filled NR nanocomposite was attributed to strain-induced crystallization of NR accelerated by well-dispersed NC. However, the larger tensile modulus and low strain for the CNF-filled NR were related to the formation of immobilized NR at the interface between CNF aggregate and NR. The immobilization effect of NR at the CNF surface offered by a mutual entanglement of CNF aggregate and NR chain led to local stress concentration and accelerated strain-induced crystallization of CNF/NR nanocomposite. From the present study, the NR nanocomposites combined with 5 phr CNF shows high-tensile modulus and acceptable breaking tensile stress and strain, suggesting the application of CNF/NR based nanocomposite in automotive and stretchable sensors for next-generation electronic devices.

## 1. Introduction

Natural rubbers (NR) are flexible materials widely used in a variety of applications due to their unique contributions, i.e., tires, dipped goods, adhesives, rubber thread, foam, conveyor belts, hoses, gaskets, footwear, and engine mounts [1]. However, their practical applications are sometimes limited due to a trade-off in their physical properties. Therefore, the introduction of new natural rubber-based products for engineering applications is challenging. One of the classical ways to achieve property improvement of NR is simply accomplished by developing multifunctional rubber composites based on NR [2,3,4].

Rubber nanocomposites are defined as two-phase systems consisting of rubbers and nano-fillers, of which at least one dimension is in the nano-range (1–100 nm) [5]. Due to their nanometer scale and high aspect ratio, well-dispersed nano-fillers within a rubber matrix result in a huge amount of surface area exposed to the rubber molecules when compared with conventionally micron-sized fillers; thus, the molecular interaction between the nano-filler and rubber matrix is increased, and further enhancement in the mechanical and physical properties of rubber nanocomposites is obtained [6]. Another aspect that warrants the use of nano-filler as an additive to rubber matrix is that the loading requirement is relatively low (1–10 phr), in contrast to a conventional filler (50–60 phr).

Nanoclay (NC) has been widely used for the preparation of rubber nanocomposites. The addition of nanoclay caused a significant improvement of NR properties, including increased strength, modulus [7,8], thermal stability [9], barrier property [10], and decreased flammability [9]. Several research works have also demonstrated that the low addition level of nano-fillers greatly improved the physical and mechanical properties of various plastics and rubbers [11,12,13,14,15,16]. In addition, when a well-dispersed nano-filler was achieved, the reinforcement of rubbers was remarkable [17,18,19]. Recently, the cellulose nanofiber (CNF) derived from plants has increasingly received numerous attention from researchers and industries due to their unique properties, i.e., high crystallinity, high modulus, high tensile strength, biodegradability, renewability, and non-toxicity [20,21,22,23]. Because of their superior properties, it has often been used as reinforcement to replace non-renewable fillers in various polymers [24,25,26]. Most studies found that the low CNF loaded polymer composites showed enhancement of specific tensile modulus and strength, contrarily to highly filled conventional polymer composites [27,28,29].

It is well accepted that the principal reason for the excellent properties and crack resistance of crosslinked NR was attributed to its ability of strain-induced crystallization [30,31]. During the deformation of crosslinked NR, the strain-induced crystallization of NR was explained by the molecular alignment of stretched chains between the dense network points, which then formed crystallites [32]. Upon nano-filler inclusion, the evolution of NR structure under tensile stretching and its crystallization behavior quite differed from those of unfilled and even conventional filled NRs [33,34,35,36]. In the case of nanoclay/NR nanocomposites, Masa et al. [13,37] showed an interesting behavior of nanoclay in the strain-induced crystallization process of NR. They proposed a model of interpreting the role of nanoclay on the strain-induced crystallization process and reinforcement mechanism of NR through the help of wide-angle X-ray diffraction (WAXS) and small-angle X-ray scattering (SAXS) analyses. Based on their model, the incorporation of nanoclay provided an early onset and enhanced ability of strain-induced crystallization under uniaxial deformation, leading to a significant increase in mechanical properties of the NR nanocomposites. As for CNF/NR nanocomposites, although several reports are showing an advantage of using CNF as a reinforcing filler in NR nanocomposites, the study on strain-induced crystallization of CNF/NR nanocomposites has never been reported, and reinforcement of NR by CNF is not clearly understood.

Therefore, the present study aimed to investigate the structure and property of nanocomposites obtained from NR and two kinds of nano-fillers, i.e., NC and CNF. The NR nanocomposites were prepared by a latex-mixing method, as previously described in the literatures [13,37]. Latex mixing is preferred because it offers efficient dispersion of nano-filler within the rubber matrix. The NR nanocomposites were then crosslinked with dicumyl peroxide (DCP). The pure NR was also prepared for reference. The dispersion of NC and CNF was examined by transmission electron microscopy (TEM) and atomic force microscopy (AFM). The mechanical properties, bound rubber content, and strain-induced crystallization of the NC/NR and CNF/NR nanocomposites were investigated by tensile test, dynamic mechanical analysis (DMA), bound rubber content measurement, and wide-angle X-ray diffraction (WAXS), respectively. Finally, the systematic comparison between NC and CNF addition on the structure–property relationship of NR nanocomposite was discussed. 

## 2. Materials and Methods

### 2.1. Materials

High ammonium NR latex-containing dry rubber content (DRC) of 60% was supplied by Yala Latex Co., Ltd. (Yala, Thailand). Sodium montmorillonite (Na-MMT or nanoclay, Kunipia-F^®^) was kindly provided by Kunimine Industries Co., Ltd. (Tokyo, Japan). Cellulose nanofiber (CNF, Nanoforest-S) made from wood pulp by using the aqueous counter collision (ACC) method was kindly supplied by Chuetsu Pulp and Paper Co., Ltd. (Tokyo, Japan). Dicumyl peroxide (DCP) was manufactured by Wuzhou International Co., Ltd. (Liaoning, China). 2,2,4-trimethyl-1,2-dihydroquinone (TMQ) was supplied by Lanxess AG (Cologne, Germany). Paraffinic oil (white oil grade A, no.15) was provided by China Petrochemical International Co., Ltd. (Shanghai, China).

### 2.2. Preparation of NC/NR and CNF/NR Nanocomposites

**Figure 1:**
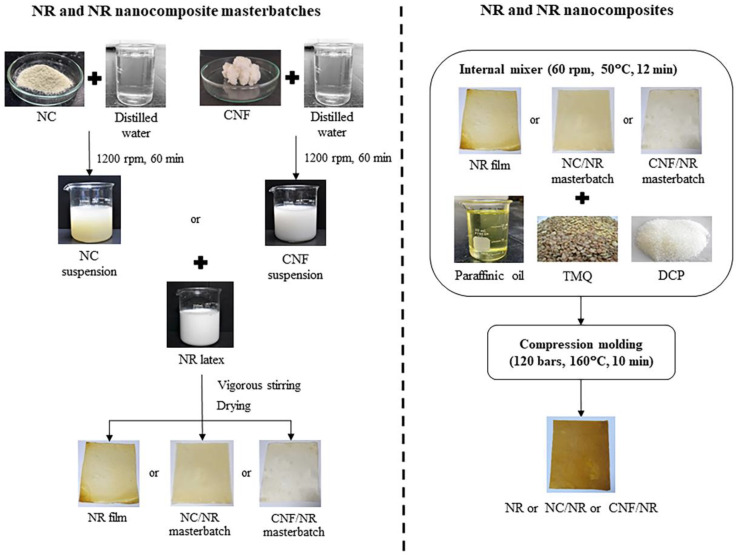
Schematic diagram of the preparation of NR and NR nanocomposites.

Two different kinds of NR nanocomposites were prepared via the masterbatch mixing approach, as schematically represented in Figure 1. The preparation involved the fabrication of NR nanocomposite masterbatch in NR latex and compounding of the NR nanocomposite masterbatch along with additives in an internal mixer, followed by a crosslinking process. In the masterbatch preparation, aqueous suspensions of nanoclay (2 wt%) and CNF (1 wt%) were firstly prepared by dispersing NC and CNF in water using IKA^®^ RW 20 digital mixer (Cologne, Germany) at 1200 rpm for 60 min. The NC and CNF suspensions were then added into NR latex and adjusted to have a final nano-filler concentration of 5 phr, and they were mixed under vigorous stirring (600 rpm) at ambient temperature for 30 min. The mixtures were dried at a temperature of 50 °C for 3 days to obtain NC/NR and CNF/NR masterbatches. From our preliminary study, the drying process provided better nano-filler dispersion, but the use of a flocculating agent to coagulate the latex mixtures caused the nano-filler aggregation. Thus, a drying process is preferred and employed in our study. Furthermore, the success of the preparation of NR nanocomposite masterbatch via the casting of aqueous suspension was reported [38,39]. The NR latex without nano-filler was also prepared using the same method and used as a control for comparison purpose. In the compounding step, the NR, NC/NR nanocomposite, and CNF/NR nanocomposite masterbatches were melt-mixing with additives in an internal mixer at a temperature of 50 °C and rotor speed of 60 rpm for 12 min. The formulation of NR and NR nanocomposite compounds is given in Table 1. The compounds formulated in this study were designed for rubber industrial manufacturers. Due to high melt viscosity of NR, the dispersion of additives and nano-fillers in the NR was limited during the mixing process; therefore, high energy is required for compounding to achieve a good additive/ nano-filler dispersion. For this purpose, the processing oil (20 phr paraffinic oil) was added to the compounds to reduce the viscosity of NR and NR masterbatches during mixing. The compounds were then compressed under the temperature of 160 °C and pressure of 120 bars for 10 min to obtain peroxide-crosslinked NR and NR nanocomposite films. In this study, the peroxide-crosslinked NC/NR and CNF/NR nanocomposites were designated as NC/NR and CNF/NR, respectively.

### 2.3. Characterizations

#### 2.3.1. Transmission Electron Microscopy (TEM)

Investigation of nano-filler dispersion in the NR nanocomposite samples was carried out by using transmission electron microscopy (TEM) (JEOL JEM 2010, JEOL Co., Tokyo, Japan). The ultra-thin section (ca., 100 nm) was cut with a diamond knife at a temperature of −120 °C by using an ultramicrotome (RMC MT-XL, RMC Products Group, Ventana Medical System, Inc., Oro Valley, AZ, USA).

#### 2.3.2. Atomic Force Microscopy (AFM)

Atomic force microscopy (AFM) was used to study the dispersion/distribution of CNF in the CNF/NR nanocomposites. The AFM images were made with a Multimode AFM (Veeco/Digital Instruments, Santa Barbara, CA, USA) with a Nanoscope IIIa controller in tapping mode at room temperature. A diamond knife was used to cut an inner part of the sample and place it on the mica surface. The silicon nitride cantilever with a spring constant of 40 Nm^−1^ was used. The scan rate of 1.0 Hz and 512 lines per 5 µm was used for optimum contrast. 

#### 2.3.3. Wide-Angle X-ray Scattering (WAXS) Measurement

The degree of crystallinity in the NR and NR nanocomposites during tensile deformation was examined by In-situ stretching Wide-Angle X-ray Scattering (WAXS) at the beamline system of BL1.3W: SAXS/WAXS Synchrotron Light Research Institute (Public Organization) (Nakhon Ratchasima, Thailand). The X-ray probe was Rayonix LX170-HS with an X-ray energy of 9 keV (wavelength of 1.3776 angstroms). The WAXS pattern was measured for every sample stretch at 20 mm. The exposure time and temperature for WAXS measurement were 30 s and 23 °C, respectively. Data were analyzed with the SAXS Image Tools (SAXSIT) program, a self-developed program for small-angle X-ray scattering [40]. The crystallinity, *X_c_*, of each sample was calculated from the intensity data of its equatorial 2-theta scan using Equation (1);

(1) $$X_{c}=\frac{A_{c}}{A_{c}+A_{a}}\times 100\%$$

when *A_c_* assigns to the area of the crystalline region and *A_a_* corresponds to the amorphous region

The orientation parameter (ƒ) of the crystal corresponding to 200 planes of various samples during stretching was estimated by using the Hermann orientation parameter, as shown in the following equation [41];

(2) $$f=\frac{3(\cos^{2}\Theta )-1}{2}$$

$$(\cos^{2}\Theta )=\frac{\int_{0}^{\pi /2}I_{c}(\Theta )\cos^{2}\Theta \sin\Theta d\Theta }{\int_{0}^{\pi /2}I_{c}(\Theta )sin\Theta d\Theta }$$

where Θ is the azimuthal angle from the stretching direction and *I_c_*(Θ) is the diffraction intensity of the crystal component at Θ. 

#### 2.3.4. Mechanical Property Measurement

The mechanical properties were performed on Hounsfield Tensometer (H10KS, Hounsfield Test Equipment Co., Ltd., Surrey, UK) at a temperature of 25 ± 2 °C with an extension rate of 500 ± 50 mm/min by ASTM D412. The dumb-bell shape specimens were cut from the crosslinked rubber films. An average of ten specimens were considered for the tensile test.

#### 2.3.5. Dynamic Mechanical Analysis (DMA)

Dynamic mechanical properties of the NR and their nanocomposites were measured by using an advanced rheometric expansion system rheometer (model ARES-RDA W/FCO, TA Instruments Ltd., New Castle, DE, USA). The storage modulus (E′), loss modulus (E″), and loss factor (tan δ) were determined with tension mode at a temperature scanned from −95 °C to 80 °C using a heating rate of 2 °C/min, frequency of 1.0 Hz, and the dynamic strain amplitude was 0.5%.

#### 2.3.6. Bound Rubber Content Measurement

Bound rubber content measurement was performed to determine physical linkages between rubber and nano-filler. About 0.2 g of uncrosslinked rubber compounds contained in the metal wire cage was immersed in 20 mL toluene at room temperature for 3 days, replacing the solvent every day. Then, the samples were removed from the toluene solvent and dried at 105 °C until they reached a consistent weight. The bound rubber content was estimated using the following equation [42];

(3) $$\mathrm{Bound}\;\mathrm{rubber}\;(\%)=\frac{W_{fg}-W_{f}}{W_{p}}$$

where *W_fg_* assigns to the weighted sample after immersion, *W_f_* is the weight of nano-filler in the specimen, and *W_p_* refers to the weight of NR in the specimen.

## 3. Results and Discussion

### 3.1. Dispersion of NC and CNF in NR Nanocomposites

**Figure 2:**
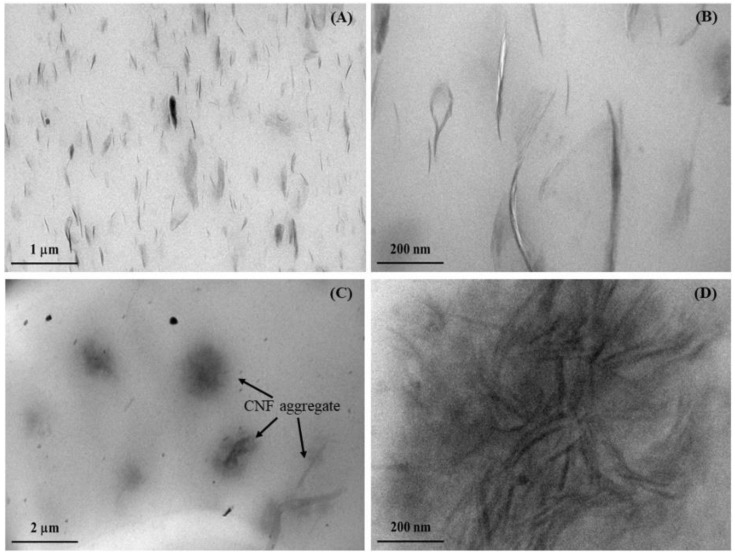
TEM images of (**A**–**B**) NC/NR and (**C**–**D**) CNF/NR taken at low magnification (**left**) and high magnification (**right**).

The dispersion state of NC and CNF at an equal amount (5 phr) in a natural rubber matrix was studied, and the results are shown in Figure 2, which consists of TEM photomicrographs of NC/NR and CNF/NR nanocomposites taken at low and high magnifications. From Figure 2A, the TEM images observed at low magnification showed the NC particles (dark lines) dispersed in the NR matrix. Upon enlargement in Figure 2B, it was apparent that a lot of NC platelets were arranged in small particles of nanoclay stacks (or tactoids) with a thickness of several nanometers, and a single platelet of 1 nm thick was not seen in the NC/NR sample. The dimension of dispersed NC particles in the NC/NR was also measured by Image J software, and the results are given in Table 2. The thickness of nanoclay tactoids was in the range of 10–40 nm. The nanoclay dimension was in agreement with the results reported by Masa et al. [37]. Figure 2C shows the dispersion of CNF in the NR matrix. It is seen that CNFs were aggregated, and the dimension of aggregated CNF was about 1–3 µm with the average value of 1.7 µm (Table 2). When the microstructure of CNF aggregates was magnified in Figure 2D, the entanglement of nanofibers was clearly observed. The CNFs have a large number of hydroxyl (–OH) groups present on their surfaces [43]. These surface –OH groups of CNFs interacted by hydrogen bonding, resulting in the formation of entangled nanocellulose fibers within the NR matrix. Although both nanoparticles were added into the NR at similar loading, the variation in dispersion between NC and CNF was obtained. This could be attributed to dissimilarity in their geometry. The NC is a rigid platelet-like nanoparticle [7], whereas the CNF is a long flexible nanoparticle [22,25]. The possibility of entanglement of CNF was then greater when compared with NC. 

**Figure 3:**
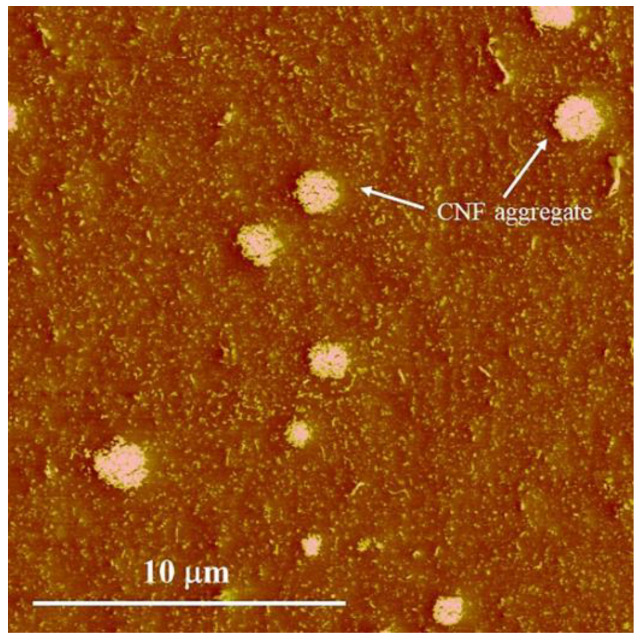
AFM image of CNF/NR observed across large area (20 μm^2^).

To confirm the dispersion state of CNF in the NR, the microstructure of the CNF/NR sample was also overviewed by AFM analysis. Figure 3 shows AFM image of CNF/NR observed at a large (20 μm^2^) investigation area. The bright region over the darker background in the AFM images is the dispersion of CNF in the NR matrix. It is seen that the CNF/NR showed inhomogeneous dispersion of CNF in the NR. Most of the CNFs were dispersed in the form of different levels of aggregations. From Figure 3, considering the AFM image obtained from the investigation across a large area (20 μm^2^), the CNF aggregates were about 1–3 µm. The shape and size of CNF aggregates appeared to be comparable to those obtained from the TEM observation, as previously discussed. The TEM and AFM results suggest that the addition of NC and CNF yielded substantially different NR microstructures, with the NC being finer dispersed in the NR than the CNF.

### 3.2. Stress–Strain Behavior of NR, NC/NR, and CNF/NR

**Figure 4:**
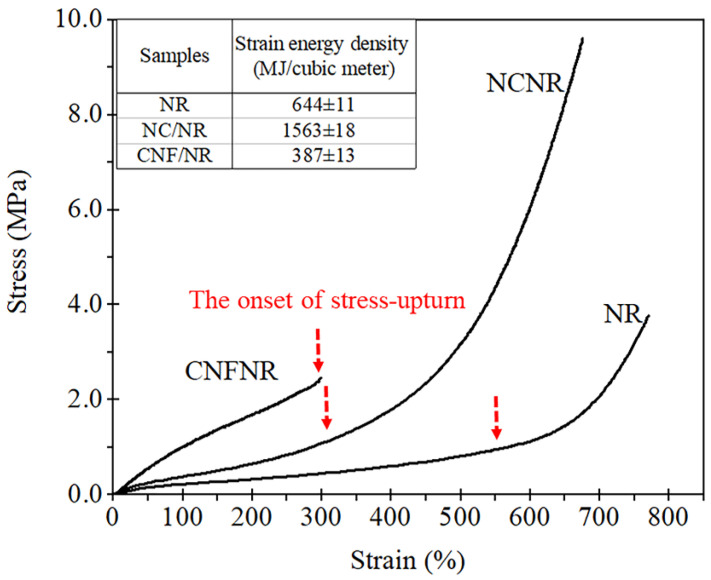
Stress–strain curves of NR, NC/NR, and CNF/NR.

Figure 4 shows stress–strain curves of various types of NR nanocomposites compared with corresponding unfilled NR. As can be seen from Figure 4, two distinctive phenomena in stress–strain behaviors could be observed. That is, the NR and NC/NR exhibited a typical strain-induced crystallization in their stress–strain curves, whereas the CNF/NR did not show any evidence of such a process during tensile testing. Generally, the stresses of NR and NC/NR gradually increased with increasing strain, followed by an abrupt increase in stress, i.e., stress upturn at a relatively larger strain. Several studies have also reported an abrupt increase in stress at the high strain in NR and NC/NR nanocomposite systems [13,34,37]. In this context, the term “stress upturn” is described as a turning point of strain, at which the stress increases sharply with applied strain. The appearance of stress upturn is ascribed to the strain-induced crystallization of NR during tensile stretching [13,37,44,45,46]. The upturn of the stress of NC/NR indicated by the arrow started to occur at a much lower strain (about 300%) when compared with that of NR (about 570%), and the ability of upturn of stress in the NC/NR was also greater than that of the unfilled NR.

The mechanical properties of the NR nanocomposites and NR in terms of 50%, 100%, and 300% moduli, tensile strength, and strain at break were also measured. The area under the stress–strain curves of NR, NC/NR, and CNF/NR were also mathematically integrated to obtain strain energy density. The data are shown in the inset table in Figure 4. The pure NR had low tensile properties, i.e., 50% modulus, 100% modulus, and 300% modulus (0.22 MPa, 0.25 MPa, and 0.49 MPa, respectively), tensile strength (3.65 MPa), but high strain at break (759%). The estimated strain energy density of NR was 644 MJ/ m^3^. As the NC (5 phr) was incorporated into the NR, the moduli at 50%, 100%, and 300% strains were a slight increase corresponding to the improvement level of about 9%, 40%, and 106%, respectively, when those of the NR were compared. The tensile strength was considerably enhanced, and the strain at break slightly decreased. The tensile strength of NC/NR was about 172% higher than that of NR. This observation was in line with the previous works [13,37]. Moreover, the strain energy density of NC/NR was about 143% greater than that of NR, implying that more energy per unit volume could be absorbed in the NC/NR before fracture. On the contrary, the tensile stress of CNF/NR was greatly increased from the low strain. However, when the strain reached a certain value (about 300%), at which the stress upturn of the CNF/NR appeared to start, the sample failed upon tensile stretching. At any applied strain, it is interesting to see that the stress of the CNF/NR was much higher than those of the NC/NR and NR. For instance, the presence of 5 phr CNF in the NR nanocomposite significantly increased the moduli at 50% strain (0.5 MPa), 100% strain (1.0 MPa), and 300% strain (2.55 MPa) by 120%, 300% and 420%, respectively. However, the tensile strength (2.6 MPa) and strain at break (300%) of the NR were markedly decreased. This was because the CNFs were interacting with each other, giving rise to relatively large aggregates (Figure 2), which acted as a stress concentration in the rubber network [47,48,49]. As a result, the tensile strength and strain at the break of CNF/NR were comparatively lower than those of NR. Siqueira et al. [24] also reported a reduction of strength and strain at break for CNF-reinforced polycaprolactone (PCL) nanocomposites due to poor dispersion of CNF in the PCL matrix. It is also seen that the addition of CNF decreased the strain energy density (387 MJ/m^3^) of the NR, meaning that the material ductility became lower. From these results, the NC/NR exhibited relatively high tensile strength and toughness, while the CNF/NR became stiff and less ductile. For NC/NR, it may be inferred that a well-dispersed NC and the mobility of NC during deformation promoted tensile strength and toughness of NR. In the case of CNF/NR, the large CNF aggregates creating a high-stress concentration field in the NR matrix could contribute to the stiffening effect of the NR, thereby making the material more fragile.

Since the crosslink density is also important property affecting the major characteristics of a crosslinked rubber, the crosslink density of the NR, NC/NR, and CNF/NR due to the crosslinking reaction between NR and DCP was also compared. The crosslink density, determined by a gel content measurement [50,51] of NR, NC/NR, and CNF/NR, was about 80.12%, 80.32%, and 80.43%, respectively. The similar gel content of NR, NC/NR, and CNF/NR reflected that the chemical crosslinking of NR and NR nanocomposites were not different, and that the change in the mechanical properties of NR (Figure 4) was predominantly related to incorporated nano-fillers.

To clearly explain the alteration in stress–strain behavior under tensile deformation for each sample and why the mechanical properties of NR were different from those of NR nanocomposites, further investigation by means of WAXS analysis was carried out, and the results were reported in the next section. 

### 3.3. Strain-Induced Crystallization of NR, NC/NR, and CNF/NR

**Figure 5:**
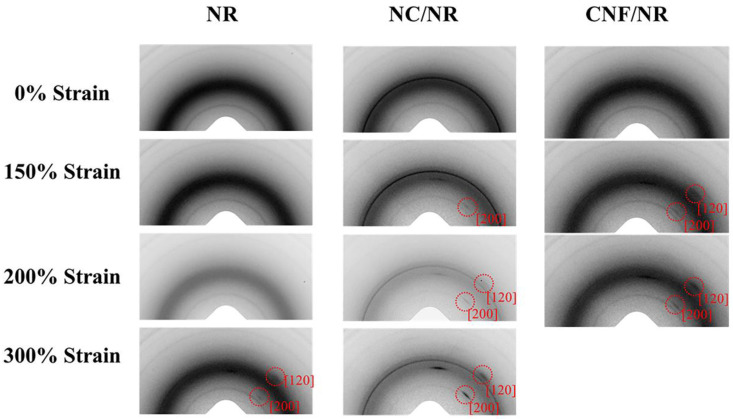
Typical two-dimensional WAXS image as a function of strain for NR, NC/NR, and CNF/NR.

Figure 5 shows two-dimensional (2D) WAXS images of NC/NR and CNF/NR at various strains. The 2D pattern of the pure NR was also included for comparison. As can be seen from Figure 5, the 2D WAXS image without a reflection spot was observed in all NR samples without tensile deformation (0% strain), implying that no strain-induced crystallization occured. Owing to highly oriented crystallite in the NR induced by stretching, the reflection spots assigned to (200) and (120) planes in the pure NR were detected at a strain of about 300%. By contrast, they were found at the strain of about 150% for the NC/NR and CNF/NR. The result suggested that the incorporation of both NC and CNF caused the early alignment of NR chains in the stretching direction, promoting the formation of crystallization in the NR matrix. 

**Figure 6:**
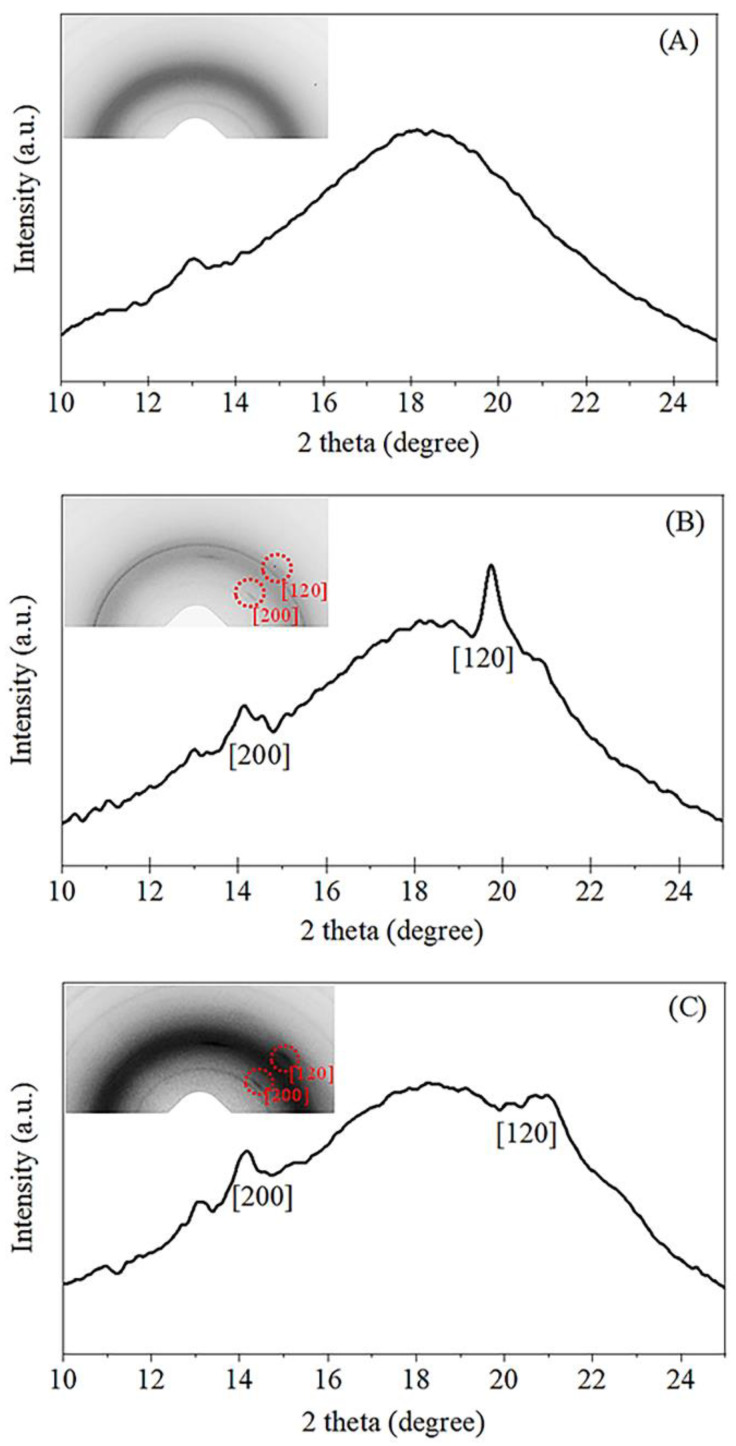
Coupled 2D WAXS images and WAXS patterns of (**A**) NR, (**B**) NC/NR, and (**C**) CNF/NR measured at a strain of 200%.

To further confirm the accelerated strain-induced crystallization in the two different NR nanocomposites, the selected 2D WAXS images shown in Figure 6 were converted to 1D patterns using the SAXSIT program. Figure 6 displays the 2D WAXS images coupled with their corresponding 1D linear patterns of the NR, NC/NR, and CNF/NR at the strain of about 200%. As previously mentioned, the pure NR sample showed no reflection spots (Figure 5); thus, its corresponding linear diffraction pattern possessed only an amorphous halo (Figure 6A). On the other hand, the highly oriented crystalline reflection spots and the crystal diffraction peaks at 2θ of about 14° and 20°, corresponding to (200) and (120) planes [52], respectively, were observed for the NC/NR and CNF/NR at the same strain (200%). The 1D WAXS patterns further highlighted that the addition of a nano-filler promoted the alignment of NR chains in the stretching direction even at low strain levels, accelerating the crystallization process of the NR matrix. 

The obtaining WAXS results were further fitted by using the SAXSIT program [33]. After peak fitting, the degree of crystallinity can be estimated by applying Equation (1). In this study, the crystallinity corresponding to the (200) plane was selected as a representative of crystallinity variation during the deformation of the NR and NR nanocomposites.

**Figure 7:**
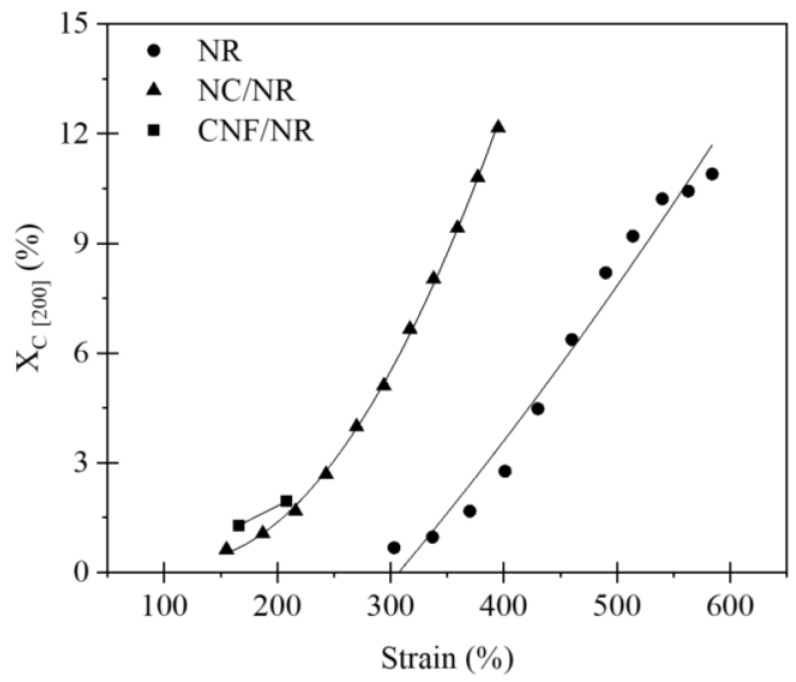
Variation of crystallinity (*X_c_*) as a function of applied strain for NR, NC/NR, and CNF/NR.

Figure 7 illustrates the degree of crystallinities (*X_c_*) of the NR, NC/NR, and CNF/NR as a function of applied strain. The crystallinity of all samples generally enhanced with increasing strain, indicating that the crystallization of NR was attributed to deformation-induced crystallization. The onset crystallinity of the pure NR sample was found at a strain of about 300%, while those of the nanocomposites were at a strain of about 150%. It has been established that the crystallization process in the unfilled NR was caused by crosslinking points and occurred most frequently at high strain (i.e., about 300%). On the contrary, the inclusion of a nano-filler could facilitate the strain-induced crystallization of the NR by lowering the strain at which the onset of crystallization occured [37]. Thus, the early strain-induced crystallization found in the NC/NR and CNF/NR was attributed to the accelerated strain-induced crystallization by nano-fillers. 

In the case of nanoclay-filled NR, the contribution of nanoclay to strain-induced crystallization and mechanical properties was well reported [13,37]. In this report, the crystallinity induced by crosslinking network structure in the unfilled NR appeared at about 300% strain, while the crystallization in the NC/NR nanocomposite was found at low strain (about 150%). It was shown that the early strain-induced crystallization in the NC/NR nanocomposite was due to nanoclay rotation during tensile deformation, generating an alignment of NR chains in the stretching direction. As a result, the evolution of crystallinity in the NC/NR was attributed to a preferred alignment of NR chains brought by nanoclay rotation at low strain levels and then induced by collaborative crystallization of nanoclay and crosslinking points at high strain levels (i.e., above 300%).

Considering the CNF/NR sample, the onset of crystallinity was also noticed at the strain of about 150%, validating the ability of CNF to cause strain-induced crystallization in the NR at low strain. Referring to Figure 2, the CNF aggregates in the NR matrix resulted in a structural defect, thereby causing a failure of the sample at low strain. Consequently, no additional crystallinity in the CNF/NR sample was developed. It is also interesting to note that the degree of crystallinity in the CNF/NR at a given strain was comparable to the NC/NR (Figure 7); however, a significant difference in stress–strain behavior was observed; that is, a stress increment of the CNF/NR was much greater than that of the NC/NR (Figure 4). The origin of these differences may be explained by the establishment of strain-induced crystallization at the interface region between CNF aggregate and NR due to stress concentration. When a critical amount of NR crystallized at the interface region, excess stress was then imposed on the surface of CNF by strain-induced crystallization. Therefore, the interface could not endure the steep increase in stress, such as stress upturn, because of lacking chemical interaction at the interface between CNF and NR. This led to the breaking of the nanocomposite sample around the interface at which the onset of stress upturn in the CNF/NR was observed. 

**Figure 8:**
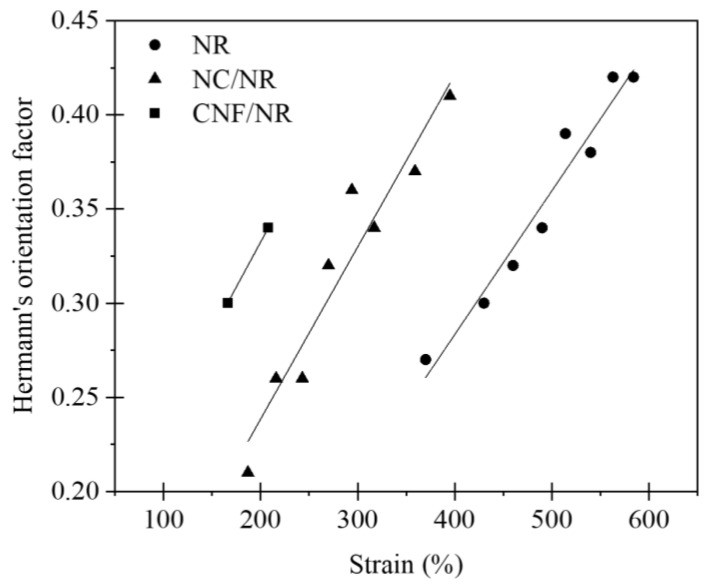
Variation of Hermann’s orientation factor (ƒ) as a function of applied strain for NR, NC/NR, and CNF/NR.

Figure 8 displays the variation of orientation parameters (ƒ) of the NR, NC/NR, and CNF/NR as a function of applied strain during deformation. It is well accepted that the ƒ value of a perfect arrangement of crystallites parallel to the stretching direction is usually 1. If the crystals have a random orientation, the value of ƒ is 0, and a value of 0.5 refers to a fully perpendicular orientation to the stretching direction [53]. From Figure 8, it is apparent that the ƒ values of all rubber samples approached 1, and their magnitude grew with increasing strains. The results implied that the crystallite orientation during stretching was nearly parallel to the stretching direction, and the degree of NR crystallite orientation was improved with applied strain. 

### 3.4. Dynamic Mechanical Properties

**Figure 9:**
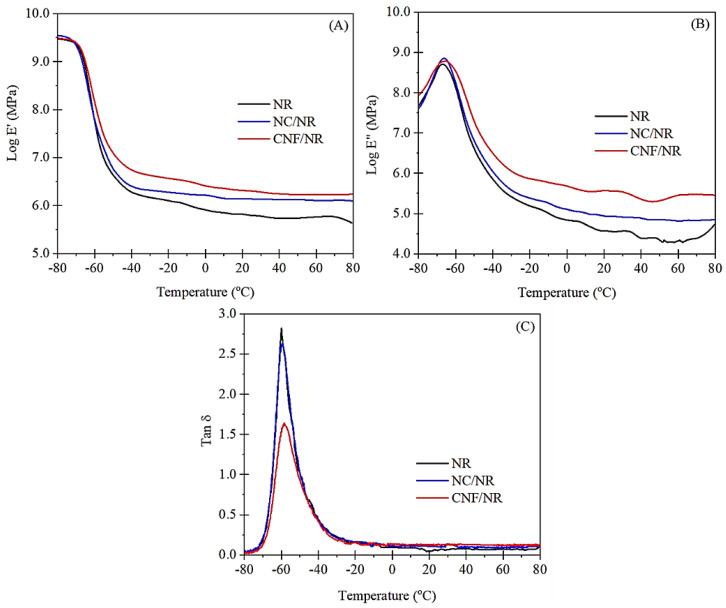
Variation of (**A**) storage modulus (log E’), (**B**) loss modulus (log E″), and (**C**) Tan δ as a function of temperature for NR, NC/NR, and CNF/NR.

The plots of storage modulus (E′), loss modulus (E′′), and damping factor (Tan δ) against temperature for the NR and their nanocomposites are shown in Figure 9. The values of E′ at 25 °C, the glass transition temperature (T_g_), and Tan δ peak height (Tan δ_max_) are summarized in Table 3. 

When compared to pure NR, the addition of both fillers increased E′ at 25 °C, and the increase was even greater when the CNF was incorporated (Figure 9A). The enhancement of E′ was attributed to the fact that the inclusion of a nano-filler into the rubber caused an increment in rubber stiffness. The larger improvement of E′ noticed in the CNF/NR was probably attributed to the dispersion of CNF with entanglement possibility, as demonstrated in Figure 2. These findings are consistent with those reported in the previous study of Azizi and co-workers [54]. They studied the mechanical properties of poly(styrene-co-butyl acrylate) reinforced with cellulose microfibers extracted from sugar beets. The cellulose microfibers were treated with sulfuric acid to obtain various fiber lengths. As the fiber length increased, the tensile modulus, as well as the storage modulus, increased due to entangling characteristics. Based on these results, the strong influence of nanofiber entanglement on the mechanical properties was the main reason for the increase in stiffness in the CNF/NR. From Figure 9, the incorporation of nano-fillers appeared to increase the area underneath the E″ peak, particularly with CNF. In general, the area underneath the maximum peak of E″ indicates dissipated energy per unit volume of the sample during the transition from glassy to a rubbery state. If rubber–filler interaction is presented in the system, fraction of the rubber chain near the interface between rubber and filler could be immobilized, and this causes the loss of energy for molecular arrangement and internal friction between rubber chain and filler [55]. Considering the glass transition temperature and Tan δ_max_ of various NR samples, the addition of nano-filler (either NC or CNF) into the NR caused a slight shift of the T_g_ toward higher temperature. The T_g_ value of NR was −60.1 °C, and it increased to −59.3 °C for the NC/NR and to −58.5 °C for the CNF/NR (Table 3). The change of glass transition temperature toward higher temperatures means that more thermal energy is required to generate rubber chain mobility [56,57]. Additionally, the Tan δ peak height or Tan δ_max_ of the NR was also decreased with the incorporation of NC and CNF. The increase in glass transition temperature and decrease in Tan δ_max_ indicated the presence of interaction between the NR matrix and nano-fillers [24,46]. This interaction would effectively restrict the mobility of the NR chains near the surface of nano-fillers, thus raising the glass transition temperature and lowering the height of the Tan δ peak. Since the increment of the area underneath the E″ peak, glass transition temperature, as well as the decrement of Tan δ_max_ were greater with the addition of CNF to the NR; thus, these observations may suggest the insertion of NR into the CNF aggregate or entangled CNF as indicated by the formation of non-extracted NR determined from bound rubber extraction experiment, which is discussed in the next section. 

### 3.5. Bound Rubber Content 

In general, bound rubber content describes the absorption of rubber onto the filler surface, depending on filler content, filler surface activity, and filler structure [58]. Table 4 summarizes the values of bound rubber content for the NR, NC/NR, and CNF/NR. 

From Table 4, it is interesting to see that the bound rubber was found only in the CNF/NR containing 5 phr CNF after immersion in toluene, while it was not seen in the NR and NC/NR with similar nano-filler loading. In the CNF/NR, the rubber attached to the CNF surface would be relatively low due to weak interfacial adhesion; however, the observed bound NR for the low filling of 5 phr CNF was significant (9%). If a large quantity of bound rubber existed at the interface between CNF and NR, the adhesion between these two phases would be strong, then the strain at break should not be much sacrificed, as shown in Figure 4. These results suggested that the relatively high bound rubber content estimated in this study contained the aggregated CNFs that were inserted with NRs. Therefore, the bound rubber content confirmed the mutually entangled CNFs and NR chains due to the insertion of NRs in the CNF aggregates. 

### 3.6. Model

**Figure 10:**
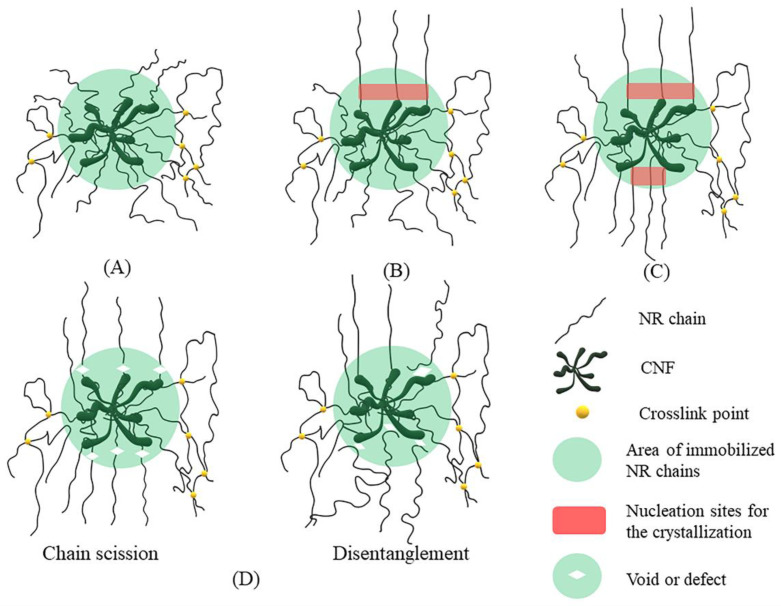
Proposed model for strain-induced crystallization mechanism of CNF/NR nanocomposite at (**A**) 0% strain (**B**) about 150% strain (**C**) about 200% strain and (**D**) over 200% strain.

To gain a better understanding of the reinforced mechanism of CNF in NR, a model explaining the reinforcement of CNF/NR during stretching was proposed, as illustrated in Figure 10. Based on the results obtained from the TEM, DMA, and bound rubber measurements, it was assumed that a portion of the NR chains was inserted in the aggregated CNFs. These inserted chains caused the formation of the immobilized chains of NR on the surfaces of CNF aggregates, as shown in Figure 10A. When the deformation started, some immobilized short chains near the surfaces of CNFs were initially stretched, and thereafter they became nucleation sites for the crystallization of NR chains when stretching to about 150% strain (Figure 10B). As a result, the highly oriented reflection spot was observed in the WAXS image of the CNF/NR sample at strain of 150% (Figure 5). The crystallization of the NR matrix was continuously increased with applying strain because the stress concentration at the interface region between the NR chains and the CNF aggregates was increased, causing further orientation and alignment of NR chains (Figure 10C), leading to the stronger oriented reflection spots due to greater sites for crystal nucleation formation during deformation to 200% strain (Figure 5). Upon applying strain greater than 200% strain, however, the excessive local stress concentration initiated the formation of voids acting as defects at the CNF/NR interface due to chain scission or disentanglement (Figure 10D). These interface voids or defects would grow larger in the sample with applied strain, and eventually, the CNF/NR failed at the strain above 200%.

## 4. Conclusions

NR nanocomposites reinforced with 5 phr nano-fillers were successfully prepared by using the latex mixing approach. The effect of nano-filler types (i.e., NC and CNF) incorporation on the reinforcement behavior was studied by means of tensile and wide-angle X-ray diffraction measurements, while the morphological, dynamic mechanical, and bound rubber formation analysis was used to evaluate the interaction between filler and rubber. It was found that the NCs with platelet morphology were uniformly dispersed throughout the rubber matrix, whereas the CNFs were aggregated and poorly dispersed. The NC was found to enhance the strain-induced crystallization of NR, leading to high tensile strength, whereas the CNF decreased the tensile strength and strain at the break of NR. However, the tensile modulus at various strains of the CNF/NR nanocomposite was significantly greater than those of the NC/NR nanocomposite and pure NR due to stress concentration at the interface between CNF aggregates and NR chains. The 50%, 100%, and 300% moduli of the CNF/NR nanocomposites were increased over those of NC/NR nanocomposites by 110%, 175%, and 150%, respectively, and NR by about 120%, 300%, and 420%, respectively. Based on the present study, it is suggested that the mutual entanglement of CNF and NR, as confirmed by TEM, DMA, and bound rubber measurement, immobilized the NR chains on the CNF surfaces, leading to local stress concentration and accelerated strain-induced crystallization of CNF/NR nanocomposite. 

## Figures and Tables

**Table 1:** Formulation of natural rubber (NR), NC/NR nanocomposite (NC/NR), and CNF/NR nanocomposite (CNF/NR).

Ingredients	Part Per Hundred Parts of Rubber (Phr)		
	NR	NC/NR	CNF/NR
NR	100	100	100
NC (Na-MMT)	-	5	-
CNF	-	-	5
Paraffinic oil	20	20	20
TMQ	2	2	2
DCP	1	1	1

**Table 2:** Dimension of dispersed NC in NC/NR and CNF aggregates in CNF/NR.

Samples	NC/NR (nm)	CNF/NR (μm)
Thickness	22 ± 15	1.7 ± 0.7

**Table 3:** Storage Modulus (log E′) at 25 °C, Maximum Tan δ Peak (Tan δ_max_), and Glass Transition Temperature (T_g_) of NR, NC/NR, and CNF/NR.

Samples	Log E′ at 25 °C (MPa)	Tan δ_Max_	T_g_ (°C)
NR	5.79 ± 0.01	2.85 ± 0.02	−60.1 ± 0.1
NC/NR	6.13 ± 0.04	2.65 ± 0.03	−59.3 ± 0.1
CNF/NR	6.30 ± 0.01	1.64 ± 0.01	−58.5 ± 0.1

**Table 4:** Bound rubber content of NR, NC/NR, and CNF/NR.

Samples	Bound Rubber Content (%)
NR	N/A
NC/NR	N/A
CNF/NR	9.06 ± 1.18

## Data Availability

The data presented in this study are available on request from the corresponding author.
